# Chemical Evaluation of African Palm Weevil, *Rhychophorus phoenicis*, Larvae as a Food Source

**DOI:** 10.1673/031.011.14601

**Published:** 2011-11-04

**Authors:** Babajide O Elemo, Gloria N Elemo, Ma Makinde, Ochuko L Erukainure

**Affiliations:** ^1^ Biochemistry Department, Lagos State University, Lagos, Nigeria; ^2^ Food Technology Division, Federal Institute of Industrial Research, Lagos, Nigeria; ^3^ School of Medicine, Ross University, Portsmouth, Commonwealth of Dominica, West Indies

**Keywords:** amino acids, edible insects, fatty acids, minerals

## Abstract

The chemical properties of the African palm weevil, *Rhychophorus phoenicis* (F.) (Coleoptera: Curculionidae), larvae were evaluated using standard methodology. The chloroform-methanol extract yielded 37.12% on a dry basis. The oil was liquid at room temperature with a flash point of 36.0 °C. Analysis of the physical constants indicated values of 192.25 Wijs and 427.70 mg KOH/g as iodine and saponification, respectively. Fatty acid analysis of the extracted oil showed the presence of unsaturated fatty acids at low levels. Palmitic acid and stearic acid constituted 35.3 and 60.5% of the oil, respectively. The usual behaviour of the oil at room temperature, irrespective of the level of unsaturation of its constituent fatty acid was noted. The total protein content of the defatted palm weevil larva (dry basis) was estimated at 66.3%. The amino acid values compared favourably to FAO reference protein, except for tryptophan, which was limiting. All the other essential amino acids were adequate. Mineral analysis revealed high levels of potassium (1025 mg/100 g) and phosphorus (685 mg/100 g). The dried and defatted palm weevil lava represents a very good source of protein, and a good complement of essential amino acids.

## Introduction

The continuous increase in population and concomitant decrease in agricultural production is are major factors leading to the grave shortfall in edible protein for consumption in developing countries; the resulting protein deficiency has been a serious cause of illness and death in many of these regions. Protein deficiency in one's diet plays a significant part in what is widely referred to as protein—energy malnutrition. In countries that suffer from widespread protein deficiency, food is generally full of plant fibers, making adequate energy and protein consumption very difficult (Schwart et al. 2003). Attempts to find new foods rich in protein is desirable, especially considering the need for new infant foods suitable for preventing protein malnutrition, and to provide a cheaper alternative source to increasingly expensive animal meal.

Insects are popular source of food in many cultures around the world, as either an occasional delicacy or as a replacement food in times of shortages, droughts, floods, or war (Sutton, 1988; De Follart 1992; Adedire and Aiyesanmi 1999). They provide a good source of proteins, minerals, vitamins, and energy, and they cost less than animal protein for poor rural communities; their consumption has averted many cases of malnutrition (Chavunduka 1975). Little is known about how to realize the full potential of insects as a food crop, particularly the edible insect species gathered in rural communities (De Follart 1992), as their nutritional and economic value are often neglected. Among such insects are larvae of the African palm weevil, *Rhychophorus phoenicis* (F.) (Coleoptera: Curculionidae).

Consumption of palm weevil is considered a delicacy among the Isoko tribe of South Nigeria. Palm weevils can be four inches long and more than two inches wide, and matured larvae are fleshy and grub-like with a high fat content. These insects are collected from the trunks of palm trees and prepared by frying in a pot or frying pan.

This study intends to give a descriptive analysis of the nutritional profile of palm weevil larva, with the aim of reporting the chemical composition of the larva assessing its nutritional properties.

## Materials and Methods

### Sample preparation

*R. phoenicis* larvae weighing a combined total of 260 g were collected from Sapele, Nigeria. They were washed and put in a vacuum desiccator for 12 hours.

### Sample analysis

Lipid extraction was carried out with chloroform/methanol solvent (2:1 v/v), and the defatted sample was analyzed for total protein and ash content according to the method of AOAC (1997). The energy content of larvae were determined with a bomb calorimeter using benzoic acid as standard (AOAC 1997).

Levels of sodium, potassium, magnesium, calcium, iron, manganese, and zinc were determined using a PerkinElmer Model 290 Atomic Absorption Spectrophotometer (PerkinElmer, www.perkinelmer.com) using standard methods (AOAC 1997). Phosphorus content was determined by the molybdovanadate solution method (AOAC 1997).

Qualitative analysis of larval amino acid content was carried out by hydrolyzing the sample with 6N HCl (AOAC 1997). Amino acids were separated in the hydrolysate with a TechniCon analyzer (TechniCon, www.technicon.com). Tryptophan content was determined by hydrolysing ∼ 100 mg of the sample with 10 mL of 4.2N NaOH at 110°C for 20 hours; analysis was done with the TechniCon analyzer (TechniCon).

**Table 1.**
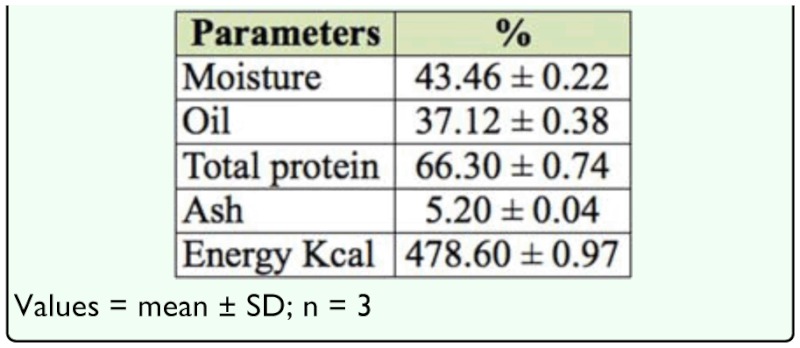
Proximate composition of the palm weevil *Rhychophorus phoenicis* larva.

Physical constant determinations of the crude oil extract, iodine number, saponification value, and peroxide value were carried out using the methods described by Pearson (1976).

Fatty acid analysis was carried out by acid catalysed trans-methylation of the lipid extract (Christie 1982). The fatty methyl esters were analysed on a Pye Unicam series 204 Gas Chromatograph (Philips, www.philips.com) with a flame ionization detector and a stainless steel column (152.4 cm and 3.17 mm id), packed with 20% diethylene glycol succinate on 80–100 mesh chromosorb for support. Column temperature was 180 °C, injection port and FID were at 210 °C, and nitrogen flow rate was set at 40 mL/min.

### Statistical analysis

Data were reported as mean ±SD. Statistical analyses were carried out using SPSS for Windows, version 14.0 (SPSS Inc).

## Results and Discussion

Over 1500 species of edible insects have been recorded in the diets of 300 ethnic groups from 113 countries. Many species of insects have served as traditional foods among indigenous peoples, and have played an important role in the history of human nutrition (De Foliart 1995). Insects have been reported to have more nutritional content than other conventional foods (De Foliart 1992). This paper reports the nutritional properties of palm weevil *R. phoenicis* larvae.

**Table 2.**
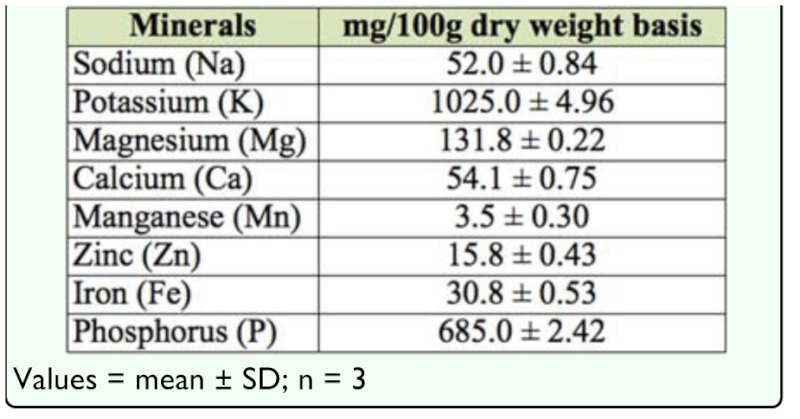
Mineral content of palm weevil *Rhychophorus phoenicis* larva.

Table 1 shows the protein, oil, ash, and energy content of the palm weevil larvae. The oil content was 37.1% while the protein of the defatted sample (dry weight basis) was 66.3%. Ash content of the defatted sample was 5.2%, which was similar to findings in Banjo et al. (2006). However, Ekpo and Onigbinde (2005) and Edijala et al. (2009) observed different results in a similar study on *R. phoenicis,* except for ash content. The energy estimate was 478.6 kcal, which is a reasonable value expected from a protein—rich sample. Edible insects have been shown to have a higher protein content, on a mass basis, than other animal and plant foods such as beef, chicken, fish, soybeans, and maize (Teffo et al. 2007). Ramos-Elorduy et al. (1997) reported the nutritional value of 78 species of edible insects in Mexico, with protein values ranging from 15–81%, and calorie content ranging from 293–762 kcal/100 g.

*R. phoenicis* larvae were found to be a very rich source of potassium and phosphorus, measuring 1025 and 658 mg/100 g, respectively (Table 2). Other minerals were found in quantities that could meet at least half of the daily recommended dietary allowance (MAFF 1985). Table 3 shows the amino acid pattern of *R. phoenicis* larvae compared favorably to egg and the FAO reference pattern (FAO/WHO 1973). The results showed that the palm weevil larvae gave efficient complementation of the amino acids, which met the FAO reference pattern with the exception of tryptophan and isoleucine. All other essential amino acids were adequate. Egg, which is considered a first class protein, had higher values of all amino acids except proline, glycine, alanine, and tyrosine. This observation corresponds to the observed high protein content (Table 1). This indicates that palm weevil larvae can act as dietary amino acid supplement in developing countries where it is readily available, in contrast with animal protein, which is quite expensive.

**Table 3.**
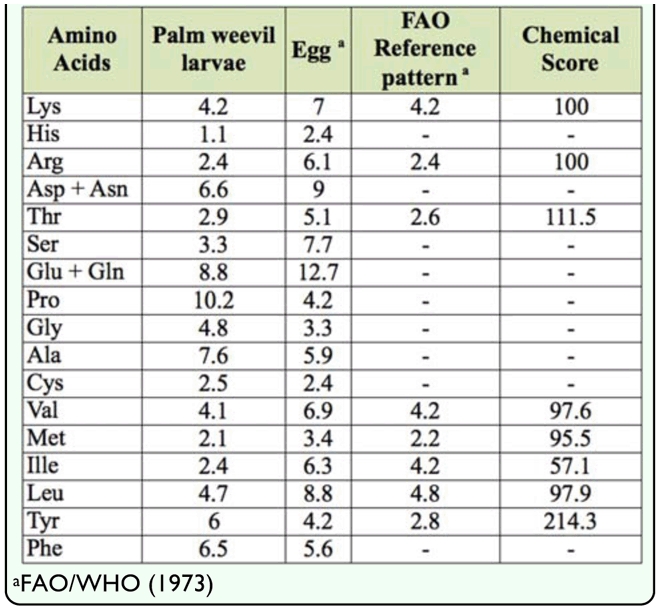
Comparative amino acid profiles of palm weevil *Rhychophorus phoenicis* larva mg/100 protein.

**Table 4.**
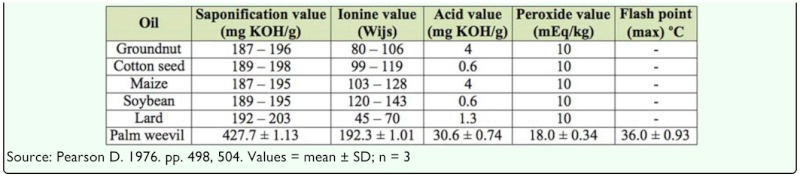
Physical constant analysis of crude *Rhychophorus phoenicis* larva oil compared to recommended codex standards.

The physical constants of the oil extract were evaluated and compared with recommended code standards for some edible oils, presented in Table 4. The palm weevil larva oil extract is unique in that it is liquid at room temperature, which is unusual for an animal oil. This may be attributed to the observed presence of unsaturated fatty acids including oleic acid (C 18:1) and linoleic acid (C 18:2) (Table 5). An iodine value of 192.3 Wijs was observed, while the saponification value was observed to be 427.7 mg KOH/g of oil. These values are relatively high when compared to that of lard and other plant oils (Pearson 1976). However, the flash point of 36 °C is within the range of lard. The high saponification value of the oil indicates its value in the soap industry (Akbar et al. 2009), while the observed high level of the acid value disqualifies the oil for use in paint and varnish industries. The high peroxide value of the palm weevil larvae oil shows that the oil is quite susceptible to oxidative rancidity (Akbar et al. 2009). However, this can be prevented and/or reduced by vacuum packing, as well as storage under cold and dark conditions that provide protection against light, oxygen, and moisture (Fernandez-Lopez et al. 2008).

Results of the percentage fatty acid composition of the oil extract are shown in table 5. The presence of the essential fatty acid, linoleic acid (3.51% of total lipid), makes palm weevil larva oil very useful in the preparation of pharmaceuticals, supporting a report by Prasad (2007) on the use of medicoe-ntomological drugs in day-to-day life in different sections of Indian society. The predominant fatty acid of palm weevil lard was stearic acid (C 18:0), measuring a significant 60.47%. Palmitic acid (C 16:0) was shown to comprise 33.3% of the total lipids, while unsaturated lipids represents only 4.23% of the total lipids. Investigations are currently underway to offer an explanation for the uniqueness in the characteristics of palm weevil larva oil extract.

**Table 5.**
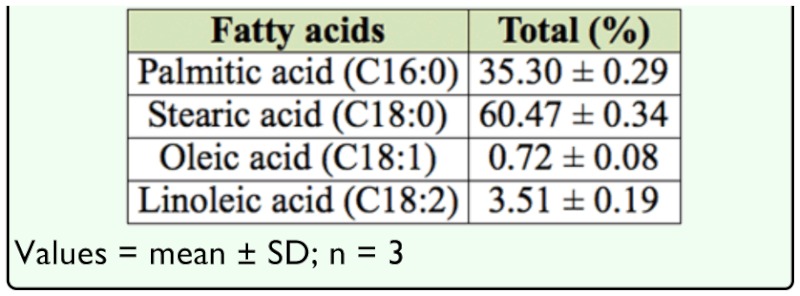
Percentage fatty acid composition of *Rhychophorus phoenicis* larva oil extract.

## Conclusion

The dried and defatted palm weevil larva compares favorably to egg protein as a very good source of protein and a good complement of essential amino acids. Further toxicological studies on the larvae of the palm weevil will prove very important in the justification and recommendation of its use in weaning foods. The extracted oil could also be used for other culinary practices and pharmaceutical preparations.
